# Multiple gouty tophi in a six-year-old

**DOI:** 10.4314/gmj.v58i3.10

**Published:** 2024-09

**Authors:** Imri G Adefokun, Gbemi H Ano-Edward, Stephen A Adesina, Peter K Uduagbamen, Samuel U Eyesan

**Affiliations:** 1 General Surgery Unit, Department of Surgery, Bowen University Teaching Hospital, Iwo, Osun State, Nigeria; 2 Anatomic Pathology, Department of Pathology, Bowen University Teaching Hospital, Iwo, Osun State, Nigeria; 3 Department of Family Medicine, Bowen University, Iwo, Osun State, Nigeria; 4 Division of Nephrology and Hypertension, Department of Internal Medicine, Bowen University, Iwo, Osun State, Nigeria

**Keywords:** gouty tophus, subcutaneous swellings, excision biopsy, pre-school, multiple swellings

## Abstract

**Introduction:**

Gouty tophus in a child is an extremely rare presentation. Only very few cases have been documented in literature in contemporary times.

**Case presentation:**

We present this index case of a 6-year-old child who was brought to the clinic by her parents on account of multiple subcutaneous swelling of two years' duration on her lower limbs before she presented at our outpatient clinic. The swellings started from the knee joints and were associated with difficulty in walking. A provisional diagnosis of multiple soft tissue swelling was made before some of the swellings were excised. An excisional biopsy of some of the masses on the lower extremities was done, and histological examination revealed gouty tophus. She was then placed on oral febuxostat. Her clinical condition has improved significantly; she is on continuous follow-up at our facility's paediatric orthopaedic outpatient clinic. Hitherto, gouty tophus has been recorded in juveniles and young adults, but it may present in any child below the age of five years.

**Conclusion:**

A high index of suspicion is needed in managing subcutaneous swellings in the paediatric age group (particularly pre-school) to identify and manage gouty tophus early enough to minimise its complications.

**Funding:**

None declared

## Introduction

Gouty tophus in preschool-aged children is extremely rare; hence, no cases have been reported in contemporary African times. A nationwide survey in Japan in 2014 found seven cases in a 2.3 million population under 15.^1^ In Korea, 2-3 cases/per 100,000 children less than 10 years of age were reported between 2007 and 2015, and in the United Kingdom, in an adult population of 502,296 less than 25 years old, only 13 cases were recorded between 1990 and 1999.^2^,^3^

Primary and secondary gout in children are two recognised forms, with primary gout more likely to be associated with lower serum uric acid levels when compared with the secondary type.^4^ Various aetiological factors have been implicated in gout in children, as some have been classified as idiopathic. Gout has also been reported in individuals with normal serum uric acid levels.^5^

First reported by Vining and Thomson in 1934, in a 5-year-old in a family with a history of gout and leukaemia.^6^ It has also been associated with various haematological and genetic conditions. 7

Apart from juvenile gout, commonly seen from age 10 upwards, gout is essentially a disease of adults.^8^ Juvenile gout could present first with symptoms under 5 years of age. Despite the inflammatory injury associated with hyperuricaemia and gout in preschool-age children, literature concerning this condition is scarce in sub-Saharan Africa. We present the first case of multiple gouty tophi in a six-year-old female in Africa.

## Case Presentation

**Figure 1:**
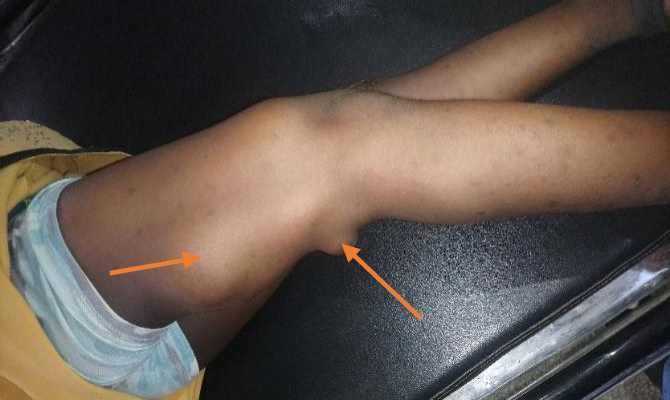
Masses on the right thigh

**Figure 2:**
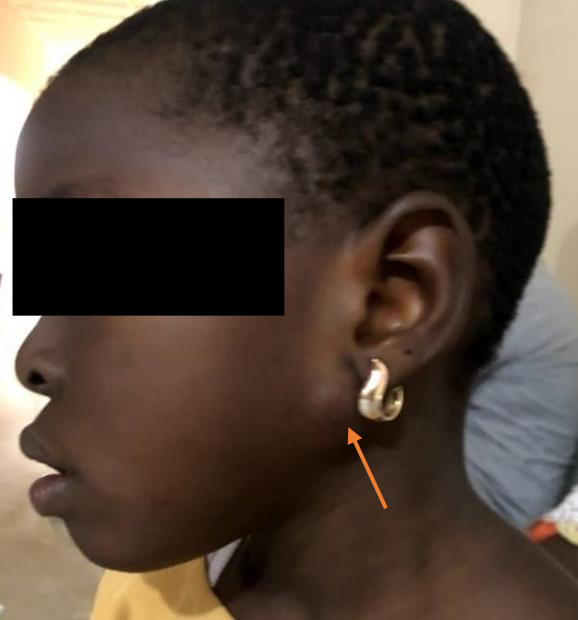
Mass at the left parotid angle

A 6-year-old girl was brought to our facility by her parents because of several hemispherical swellings on her body that had been present for two years (Figure 1). The swellings, first noticed by the mother, were initially small but progressively increased to present dimensions of 8cm (thigh) and 2cm in other parts. The swellings first appeared on both knee joints, then on the thighs and shin of the legs and left parotid angle (Figure 2).

The swellings were associated with pain and limited the patient's movement. The child was given analgesics (paracetamol and ibuprofen syrup) and antibiotics (amoxicillin syrup) at the local health centre where they lived without any resolution. There was no history of failure to thrive (FTT), intellectual incapability, or self-mutilation. The index patient was fully immunised by the Nigerian childhood immunisation schedules. There was no history of chronic cough, night sweats, weight loss, trauma to swelling sites, bleeding from the gums, or fever. The index patient is the third child of her parents, and there was no similar illness among family members.

**Figure 3:**
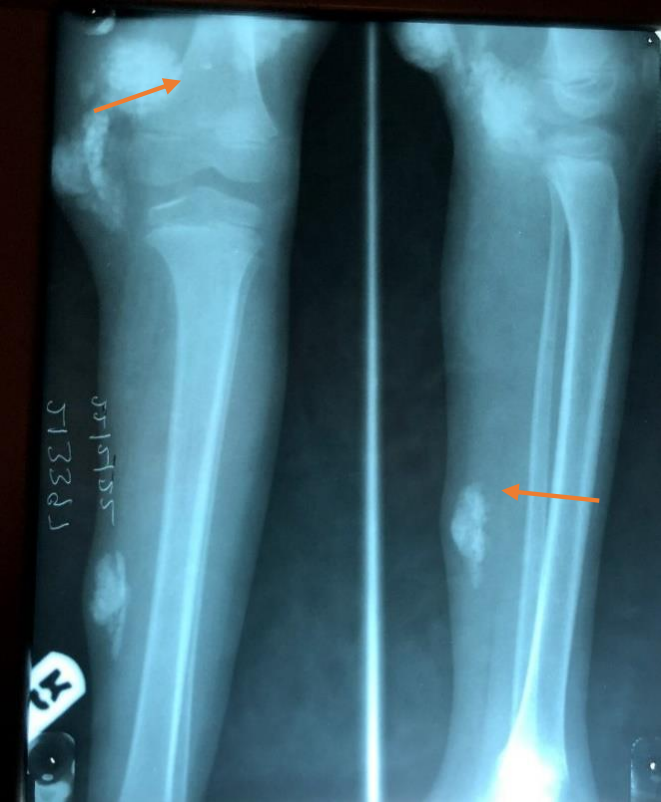
X-ray of thigh and shin; orange arrows showing tophaceous deposits

She looked well. Her height and weight were 107 cm and 18 kilograms, respectively. She had 21 multiple swelling points on both lower limbs and the face (Figures 1 and 3). The masses were firm, non-tender, and mobile. They weren't attached to the overlying skin or underlying structures, had no differential warmness over them, and ranged from 2x2cm to 8x6cm in size. The knees are deformed and asymmetrical.

**Initial Assessment:** Septic arthritis with multiple abscesses in both lower limbs. Laboratory findings include haematocrit – 35%; leucocytes - 8.4x10^9^/L (N-66%, L-27%, M-7%), and platelets – 446x10^9^/L Serology was negative for hepatitis B virus (HBV) and HIV. She was blood group A Rhesus Positive and hemoglobin Electrophoresis showed AA. She had X-rays of both lower limbs (Figure 3).

**Radiological report:** There are multifocal soft tissue density lesions ranging from medium to calcific range extending along the thighs, around the knee region, and mid to distal third of the legs, varying in dimensions, suggestive of tophi. There are subcortical intraosseous calcific lesions surrounded by an area of lucency around the femur of both thighs across the lateral aspect of distal metaphysical through the physical plate of the femur. No periarticular lucency. The femuro-tibial joint space is preserved, but some calcifications are seen posterior to the joint space. Overall features are highly suggestive of gouty arthritis with extensive tophaceous deposits.

Fine needle aspiration cytology (FNAC) was inconclusive, but an excisional tissue biopsy of one of the masses over the anteromedial part of the left knee was carried out and sent for histology.

**Figure 4:**
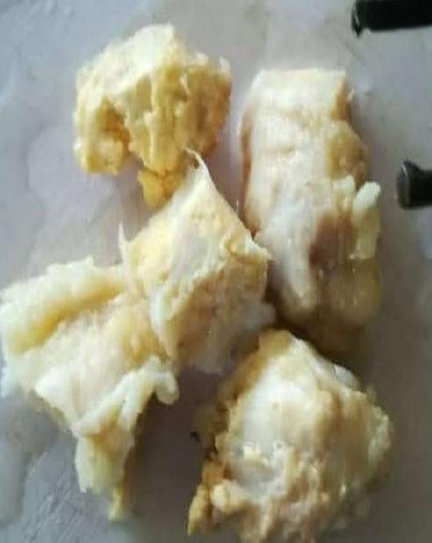
Dissected gross sample of excised lesion

The histopathology laboratory received 2 pieces of firm grey-yellow (chalky and creamy in colour) tissue measuring 7x7x3cm and 4x2x1cm (Figure 4).

**Figure 5:**
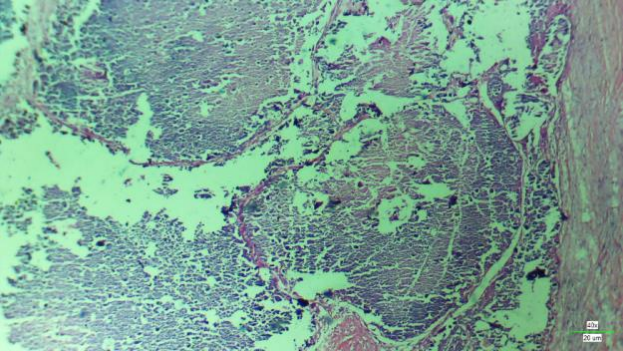
Micrograph of gouty tophus X 40 Mag

Histological findings: white crystals walled off by fibrous bands of tissue, giving a nodular appearance. Occasional benign giant cells with multiple nuclei, few lymphocytes, and fibroblasts were demonstrated. The pathological diagnosis was gouty tophi (Figure 5)

Other laboratory findings were: serum urea – 25mg/dL (Reference range{RR}: 8.0-35mg/dL) and creatinine 0.7mg/dL (RR: 0.4-1.1mg/dL) assessed. Serum uric acid was elevated at 7.2mg/dL (2.0-5.4mg/dL), calcium 9.1mg/dL (8-11mg/dL), phosphorus 6.6mg/dL (RR: 2.0-7.0mg/dL), Alkaline phosphatase (ALP) 28IU/L (36-96IU/L).

She was commenced on oral Febuxostat 20mg daily. The serum uric acid at the follow-up visit was normal (2.79 mg/dL). The masses taken from the contralateral lower limb (which had limited her mobility) during a planned second surgery were also histologically diagnosed as gouty tophus. Genetic studies were not conducted as they weren't available in our facility. Moreover, the patient had no symptoms or signs of any congenital anomaly.

## Discussion

Gouty tophi are pathognomonic for advanced gout and are characterised by nodular swellings in soft tissues. The masses are composed of precipitated urate crystals resulting from chronically elevated serum urate levels that have been untreated for several years, usually over 10 years.^9^ This index case disagrees with the common pattern as the patient presented at six years old, having developed symptoms at four years old.

The multiple-site affectation agrees with previous findings of gouty tophus in virtually any tissue, including intra-articular or extra-articular locations in the ear auricle, particularly the helix. 10 The multiple-site affectation could be of cosmetic, psychological, and clinical importance as was in the index case, in which the closeness of the masses to some major blood vessels prevented their excision. Surgery, as was done in this index case, remains a significant aspect of the treatment modality, particularly with painful masses. Surgery is also useful in preventing and treating skin necrosis, wound ulceration, infection, tendon impairment, nerve compression, and joint deformity.^11^ This was evidenced by the significant clinical improvement shown by the patient. The benefits of surgery in management also informed our decision to excise the lesion on the face during her late childhood/teenage years.

The pharmacological treatment of hyperuricemia with Febuxostat in the index case was based on the inability to excise all the masses coupled with the child's age and normal renal biochemistry to prevent the complications of chronic hyperuricemia. We considered the possibility of a background cause of hyperuricemia in the patient. However, the lack of facilities in our centre and her parents' financial status limited further investigations.

The treatment offered so far and the clinical response have been impressive, as no new swellings or increases in the sizes of the masses not excised have been noticed.

### Ethical Consideration

Ethical forms were duly obtained from Bowen University Teaching Hospital's ethical committee with Registration number NHREC/12/04/2012 and Approval number BUTH/REC-778. Obtained 8^th^ June 2023

### Consent Form

The consent form was filled and signed by the child's parents after explaining to them in detail the nature of the publication.

## Conclusion

Although extremely rare, gouty tophi in a preschool child should be considered a differential diagnosis of any soft tissue swelling in children. Urate-lowering drugs and surgery, where indicated, have immense benefits and can restore normal joint function and improve the quality of life of the children, as was the case in the index patient.
